# Development of stable transgenic maize plants tolerant for drought by manipulating ABA signaling through *Agrobacterium-*mediated transformation

**DOI:** 10.1186/s43141-021-00195-2

**Published:** 2021-06-24

**Authors:** Sridevi Muppala, Pavan Kumar Gudlavalleti, Kodandarami Reddy Malireddy, Sateesh Kumar Puligundla, Premalatha Dasari

**Affiliations:** 1 Department of Biotechnology, Nuziveedu Seeds Limited, Hyderabad, Telangana 501401 India; 2 Department of Biotechnology, Jawaharlal Nehru Technological University, Hyderabad, Telangana 500085 India; 3 Department of Plant Molecular Biology, International Centre for Genetic Engineering and Biotechnology, New Delhi, 110067 India

**Keywords:** Abiotic stress, *Agrobacterium*-mediated transformation, Immature embryo, Inbred line, Maize

## Abstract

**Background:**

In crop plants, to cope up with the demand of food for rising population, revolutionary crop improvement programmes are being implemented for higher and higher yields. Abiotic stress, especially at flowering stage, causes drastic effect on yield in plants. Deforestation and urbanization made the water table very low and changed the climate which led to untimely and unforeseen rains which affect the yield of a crop through stress, both by lack of water as well as water logging (abiotic stress). Development of tolerant plants through breeding is a time-consuming programme and does not perform well in normal conditions. Development of stress-tolerant plants through transgenic technology is the better solution. Maize is a major crop used as food and fodder and has the commercial value in ethanol production. Hence, the genes *viz.*, *nced* (9-cis-epoxycarotenoid dioxygenase) and *rpk* (receptor-like protein kinase), which play the key roles in the abscisic acid pathway and upstream component in ABA signaling have been transferred into maize plants through *Agrobacterium*-mediated transformation by optimizing several parameters to obtain maximum frequency of transformation.

**Results:**

Cultures raised from immature embryos of 2-mm size isolated from maize cobs, 12–15 days after pollination, were used for transformation. *rpk* and *nced* genes under the control of *leaP* and *salT* promoters respectively, cloned using gateway technology, have been introduced into elite maize inbred lines. Maximum frequency of transformation was observed with the callus infected after 20 days of inoculation by using 100 μM acetosyringone, 10 min infection time, and 2 days incubation period after co-cultivation resulted in maximum frequency of transformation (6%) in the NM5884 inbred line. Integration of the genes has been confirmed with molecular characterization by performing PCRs with marker as well as gene-specific primers and through southern hybridization. Physiological and biochemical characterization was done *in vitro* (artificial stress) and *in vivo* (pot experiments).

**Conclusions:**

Changes in the parameters which affect the transformation frequency yielded maximum frequency of transformation with 20-day-old callus in the NM5884 inbred line. Introducing two or more genes using gateway technology is useful for developing stable transgenic plants with desired characters, abiotic stress tolerance in this study.

## Background

Unlike animals, plants are sessile and exposed to different biotic and abiotic stresses, which refers to the conditions imposed externally and cause drastic effect in their growth and development which finally influence the productivity of the plant. Most of the plants and or crops are sensitive to abiotic stresses *viz*., drought, salinity, temperatures, and other environmental extremes. For compensating such adverse conditions, plants develop unique mechanisms to react with the tough environmental conditions by changing themselves, flexible to adapt the conditions. Several molecular changes occur to control the stress conditions by controlling the mechanisms for abiotic stress tolerance by the activation and regulation of specific stress-related genes. Development of varieties with improved growth under stress conditions is a big challenging task in crop plants. Though the development of drought-resistant plant varieties is the straightforward method, it is not only the time-taking method, but also it may not perform well in the normal conditions. Molecular genetics and biotechnology play an important role to get the resistant plants either by enhancement of native genes or by introducing the resistance genes from the other plant species under the control of stress-induced promoter.

Mostly, under stress conditions, membrane transport and perception systems have critical and essential roles for maintaining the cellular homeostasis. Abiotic stress conditions such as drought induces ABA biosynthesis which initiates the signaling pathways that lead to a number of molecular and cellular responses, among which the best known are the expression of stress-related genes and stomatal closure. ABA biosynthesis and catabolism are known to be major determinants of endogenous ABA levels in plant cells [1, 2]. In ABA biosynthesis, 9-cis-epoxycarotenoid dioxygenase (*nced*) gene encodes a key enzyme which up-regulates endogenous ABA levels in over expressed transgenic plants under drought, thereby leading to lower transpiration rates [3, 4]. Over expression of *NCED* gene resulted in ABA accumulation and increase the drought tolerance in tomato [5], cowpea [6], tobacco [7], peanut [8], rice [9], petunia [10], cotton [11], and *Arabidopsis* [12].

Stress-inducible genes function not only in protecting cells by producing metabolic proteins, but also in regulating important genes for signal transduction in the stress response [13, 14]. ABA signal transduction initiates signal perception by ABA receptors and transfer *via* downstream proteins, which includes protein kinases and phosphatases. Receptor-like protein kinases (RLKs), which are receptors localized on the plasma membrane, play important roles in stress response. Transgenic plants overproducing RPK1 have an increased tolerance to drought stress as well as oxidative stress. It also controls reactive oxygen species’ homeostasis and enhances both water and oxidative stress tolerance in *Arabidopsis* [15].

Maize (*Zea mays* L.) is the principal crop of the world. Drought causes significant decrease in maize grain yield, especially at sensitive reproductive stage. *Agrobacterium*-mediated genetic transformation is one of the best tools in biotechnology for getting the plants with desired genes. Transgenic maize plants were obtained using different types of explants *viz.*, immature embryos [16], shoot apices [17], and immature embryo-derived calli [18]. Immature embryos were proved to be the most preferred explants for highly efficient transformation. Hence, the present study has been taken up to develop the drought-tolerant maize lines by introducing two genes *viz*., *nced* and *rpk* which play the key role in the ABA pathway by adopting *Agrobacterium*-mediated transformation.

## Methods

### Plant material

Commercial maize lines *viz*., NM74C and NM5884, proprietary elite inbred lines of Nuziveedu Seeds Limited, Hyderabad, were used as a source material. For ensuring the continuous supply of immature embryos (IEs), seeds were sown in 4–5 lines staggeredly with 3–4-day intervals. Strict selfing of the cobs was done.

### *Agrobacterium* strain and construct

**Fig. 1:**
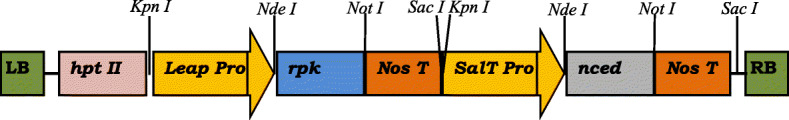
T-DNA composition of vector used in *Agrobacterium-*mediated transformation experiments of maize (*Zea mays* L.) with multi gene construction in single binary Gateway plant transformation vector *p*MDC99 having *rpk* (*receptor-*like *protein kinase* ) and *nced* (9-cis-epoxycarotenoid dioxygenase) genes driven by *LeaP* and *SalT* promoters respectively

Hyper virulent strain of *Agrobacterium tumefaciens,* EHA105, containing the gateway vector pMDC99 was used for transformation experiments. This construct contains two genes *viz*., *rpk* and *nced* driven by *Lea* (Lea P) and *SalT* (SalT) promoters, respectively. Both the genes were terminated by nopaline synthase (*nos*) terminator. Hygromycin phosphotransferase gene (*hpt II*) was used as plant selectable marker (Fig. 1).

### Explant preparation

**Fig. 2:**
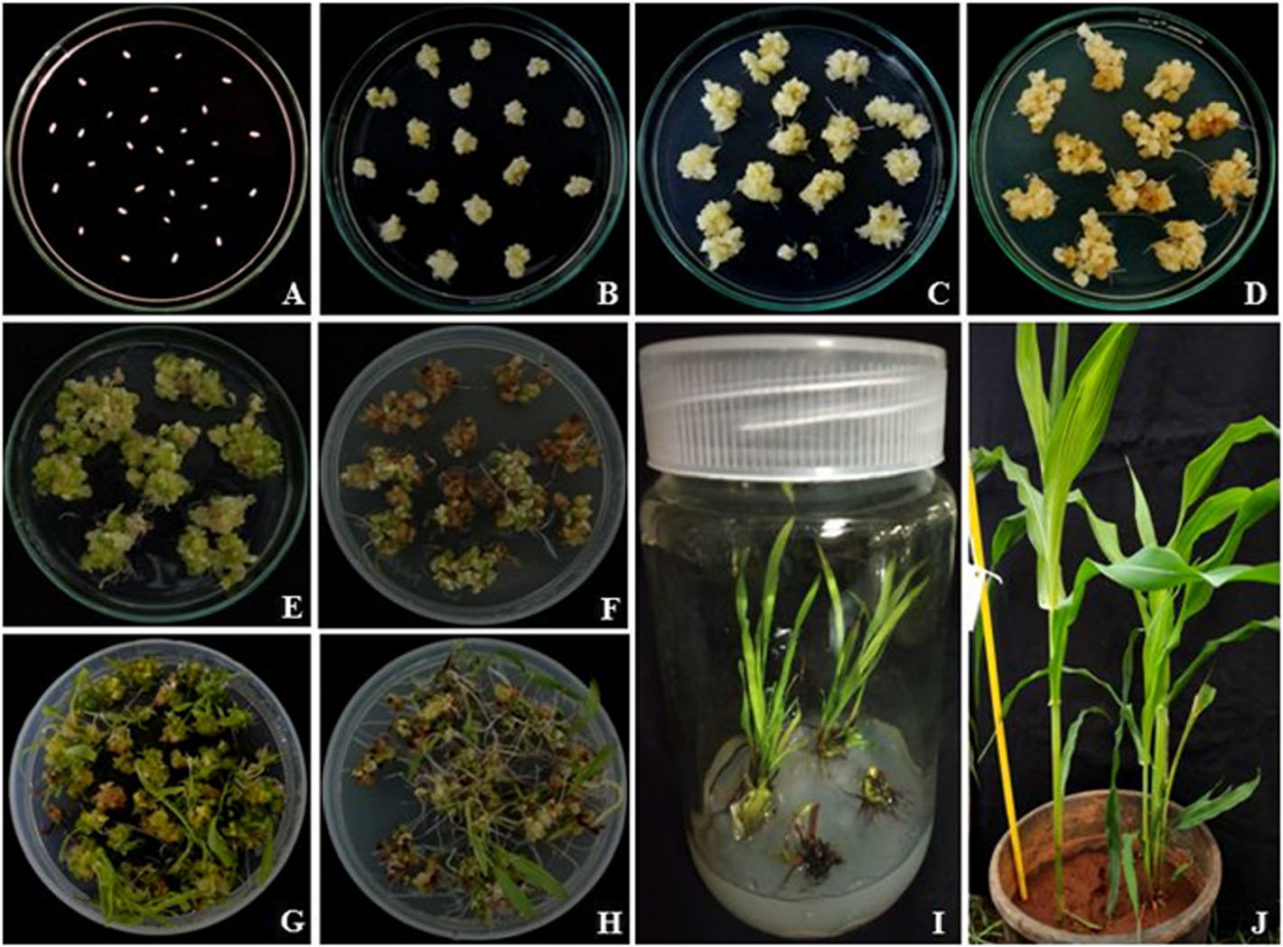
Different stages of maize cultures from inoculated nascent explants (immature embryo) (**A**) to fully developed hardened plants transferred into pots grown in poly house (**J**), after going through different stages of *Agrobacterium*-mediated transformation *viz*., co-cultivation (**B**), selection I (**D**), selection II (**F**), regeneration of plantlets (**H**), and rooting of plantlets (**I**); cultures without co-cultivation acts as controls at every stage (**C**, **E**, & **G**)

Immature ears harvested between 12 and 15 days after pollination (DAP) from the field-grown plants were sterilized with 10% sodium hypochlorite solution (4% w/v) for 15 min, followed by three washes with sterile distilled water for 5 min each. Young kernels were dissected to isolate the embryos; of which IEs of 2-mm size only used as explants (Fig. 2A).

### Infection and co-cultivation of explant

Single colony picked up from the *Agrobacterium* cultures plate was inoculated in Luria Bertani broth (LB) liquid culture supplemented with rifampicin and chloramphenicol at a concentration of 25 mg/L each and kanamycin at 50 mg/L and incubated at 28 °C by shaking at 200 rpm for overnight until it attains 0.8 optical density (OD). Then it (suspension) was centrifuged at 6000 rpm for 20 min, and the pellet was dissolved in equal volume of infection medium and then used for infecting the explants (IEs) or calli.

### Callus induction and regeneration

Linsmaier and Skoog (LS) media supplemented with a single concentration (1.5 mg/L) of 2, 4-dichloro phenoxyacetic acid (2, 4-D) was used in all steps (except regeneration) involved in transformation *viz*., infection, co-cultivation, selection, which was found to be the best concentration in our previous study [19]. LS medium supplemented with 6-benzylaminopurine (BAP) and kinetin each at a concentration of 0.5 mg/L has been used as regeneration medium.

### Selection of transformed E. calli, its regeneration, and rooting

Proliferated calli which showed resistance in two selections against 10 and 12 mg/L hygromycin respectively were transferred onto the regeneration medium with BAP and Kn each of 0.5 mg/L by maintaining in light at 26 ± 1 °C under 16/8 h. (light/dark) photoperiod. Then the regenerated shoots were transferred onto MS basal medium for elongation and rooting. Profusely rooted plantlets were maintained in cocopeat at an ambient temperature of 28 ± 1 °C for primary hardening. After 12 days, the plants were transferred onto the soil in polyhouse.

### Characterization of transgenics

#### Molecular characterization

##### PCR analysis

Genomic DNA was extracted from young leaves of putative transgenic plants along with untransformed plants (control) using the CTAB method [20]. Specific primers designed for two genes and selectable marker namely, *rpk, nced,* and *hptII* were used. The primer sequences are forward—5′CGTGGACAGCACAAGACAAC3′ and reverse—5′GTCGGAAACCCAGCTAGACA3′ for *rpk* gene, forward—5′GGTTGGTCAGATTCCTTCTTG3′ and reverse—5′GACCGTCGATCTTCACTTGG3′ for *nced* gene, and forward—5′CACAATCCCACTATCCTTCGC3′ and reverse—5′GCAGTTCG GTTTCAGGCAGGT3′ for selectable marker (hygromycin).

##### Southern hybridization

Genomic DNA (20 μg) from positive putatives along with control plants were digested with BamHI restriction enzyme overnight at 37 °C in water bath. Then the digested products were separated on 0.8% agarose gel, and the depurinated gel was transferred to positively charged Hyband-N + Nylon membrane (Amersham Biosciences, UK) by the upward capillary method described by Sambrook and Russel [21]. Then the UV cross-linked Nylon membrane was probed with Alkaphos non-radioactive direct labeling kit (Amersham Biosciences, UK) in the hybridization oven. After washing the membrane with primary and secondary wash buffers, detection reagent was applied to the membrane and finally exposed to an X-ray film. Then the film was developed for copy number enumeration. All the above procedures were followed as per the instruction manual (Amersham Biosciences, UK) supplied along with the kit. Primarily the membrane was probed with *nced* gene, after exposure, the membrane was stripped and again processed with remaining probes namely *rpk* and *hptII.*

#### Physiological and biochemical characterization

T_1_ plants which were found to be positive for selectable marker as well as two genes were selected (15 plants); five plants each from three elite lines (ET-2-1, ET-2-4, ET-2-5, ET-2-12, ET-2-15, ET-5-2, ET-5-3, ET-5-8, ET-5-11, ET-5-14, ET-9-1, ET-9-2, ET-9-7, ET-9-10, ET-9-18) were characterized physiologically and biochemically, for which ion leakage, lipid peroxidation, proline estimation, and pigment analyses (chlorophyll (Chl)-A, Chl-B, total chlorophyll, and carotenoids) were studied.

##### Artificial stress by methyl viologen

For physiological and biochemical characterization, stress has been induced by treating the leaf disc material with methyl viologen at a concentration of 250 μM, exposed for 36 h of dark continuously.

##### Electrical conductivity for measuring ion leakage

Ion leakage studies were done by following the protocol described by Kumari et al. [22]. Leaf discs, treated with methyl viologen, were washed with distilled water, and electrical conductivity (Ei) was recorded. Then the leaf discs were exposed to continuous light for 12 h, and EC (E1) was taken. Finally, the leaf discs were incubated at 100 °C for 1 h, and then E2 (final EC) was taken after cooling the tubes and ion leakage was calculated by using the standard formulae.

##### Lipid peroxidation

Lipid peroxidation was studied by measuring the MDA content by following the standard protocol described by Heath and Packer [23]. A 5% TCA (trichloro acetic acid) was used to grind the leaf material and 20% TCA containing 0.5% TBA was mixed with supernatant and incubated for 30 min at 95 °C in water bath. It was cooled immediately by keeping in ice. OD values of supernatant were taken at A_532_, A_600_, and A_450_ for calculating MDA content with standard formula by using 155/M/cm extinction coefficient, and the concentration of MDA was expressed in nM/gFW.

##### Estimation of proline content

Free proline content was estimated by following Bates et al. [24]. Leaf samples (0.5 g) were homogenized in sulphosalicylic acid (3% w/v), and homogenate was filtered through filter paper. After adding the acid, ninhydrin, and glacial acetic acid, the resultant mixture was heated for 1 h in water bath at 100 °C. Then the reaction was stopped by using an ice bath. The mixture was extracted with toluene, and the absorbance was measured at A_520_ nm. Proline concentration was determined using standard curve and expressed as μg/g tissue.

##### Chlorophyll (Chl-A, Chl-B, and total) and total carotenoid estimation

Standard procedures [25] were used to analyze photosynthetic pigment analyses. Leaf discs (transgenics as well as non-transgenic) were subjected to 36 h methyl viologen stress and incubated overnight in dark with 5 mL of DMSO for chlorophyll extraction. Then the extracted chlorophylls were estimated through photometric methods at different wavelengths of A_663_, A_645_, and A_480_ for chlorophyll-A, chlorophyll-B, total chlorophyll, and total carotenoids. Calculations were done by using standard formulae.

##### Pot experiment of transgenic maize for drought screening (*in vivo*)

Pot experiment was conducted with three independent transgenic plants (ET-2-3, ET-5-17, and ET-9-14) along with wild-type under severe water deficit condition (9 days), and then leaf samples were collected for ABA analysis. After 9 days, plants were recovered by pouring the water equally to all plants.

### Hormone (ABA) extraction and analysis

Standard procedure established by Dobrev and Vankova [26] has been followed. Leaves (100 mg) collected from control and transgenic plants subjected to drought stress were ground, by adding 5 mL of 80% cold aqueous methanol and kept in dark at 4 °C overnight. The extract was filtered twice and finally total methanolic extract was dried using N_2_ gas. The residues were dissolved in 1 mL of 80% methanol solution and vortexed. The dissolved samples were filtered and placed in vials by adjusting the volume to 1.5 mL with the mobile phase for the analysis using a high-performance liquid chromatography (HPLC) system (Waters e2695). Separation was carried out on a Waters Symmetry C18 column (4.6 × 250 mm, 5 μm) by maintaining at 25 °C with 0.5 mL/min flow rate. The mobile phase was composed of acetonitrile (60%) and 1% glacial acetic acid (40%) and performed in isocratic method. The target component was quantified by the peak areas at the maximum wavelength of 254 nm.

### Statistical analysis

Data obtained on the effects of various parameters on survival frequencies on hygromycin selection regime and physiological and biochemical data obtained from transgenic plants with reference to their control wild type plants were subjected to statistical analyses by following Snedecor and Cochran [27].

## Results

Our *in vitro* studies in maize [19] revealed that immature embryos (IEs) of 2-mm size isolated 12–15 days after pollination gives high frequency of regeneration, and the larger quantities of embryonic callus (E.callus) from the inbred lines NM74C and NM5884 among the four inbred lines studied. Hence, these two inbred lines which were found to be more regenerative and yielded larger quantities of E.callus have been used in the present study by following *in vitro* practices adopted in our previous studies *viz*., Linsmaier and Skoog’s (LS) medium [28] supplemented with 1.5 mg/L 2, 4-D for callus induction and LS with BAP and Kn (0.5 mg/L each) for regeneration [19].

### Influence of callus age on transformation efficiency

For genetic transformation, initially *Agrobacterium* infection was given to the explants (Fig. 2A), which was found to be not promising not only for callus growth and survival frequency of callus but also the plantlet regeneration. Most of the embryos did not induce any callus and turned to brown; only few embryos induced primary calli, but plantlets were not generated from them in both the genotypes; hence, the IEs with initiated callus at different time intervals after inoculation *viz*., after 10, 20, 30, and 40 days have been used for co-cultivation (Table 1). Calli which proliferated from the explants have been subcultured onto the media supplemented with hormones and cefotaxim (500 mg/L) without antibiotic (hygromycin). After 2 weeks, calli were transferred onto the selection medium I and then to selection medium II (LS medium supplemented with 1.5 mg/L 2, 4-D having 10 and 12 mg/L hygromycin respectively) with an interval of 2 weeks. The transformed calli survived on the selection medium II, have been transferred onto regeneration medium. Survival frequency of callus was studied at both the selection stages as well as regeneration stage (Table 1). IEs cultured without co-cultivation with *Agrobacterium* were served as controls throughout the experiment (Fig. 2C, E, G). Survival frequency on selection medium II ranged from 1 to 46% and regeneration frequency ranged from 1 to 25% in these genotypes. Maximum survival and regeneration frequencies of 46 and 25 were found in NM5884 inbred line where infection was done after 20 days, and lowest survival (1%) and regeneration (1%) frequencies were observed in NM74C inbred line. Co-cultivation given 20 days after explant inoculation, resulted in maximum survival frequency of callus at subculture as well as both the selection stages (Table 1) (Fig. 2B, D, F). Regeneration frequency has also been found to be maximum in both the inbred lines. Callus growth—*i.e*., survival frequency at all stages (subculture, selection I and II)—as well as regeneration frequency were found to be increased with increasing time interval of co-cultivation up to 20 days and then decreased with further increase in time in both the inbred lines (Table 1). Among the two inbred lines used, NM5884 resulted in better survival frequency of callus as well as regeneration frequency over the other inbred line NM74C in all time intervals. Variation among these two inbred lines was found to be significant (ANOVA) (*p* = 0.05) for survival frequency as well as regeneration frequency. But, these two parameters were not found to be significant in any of the inbred lines (within the group variation) (*p* < 0.05).

**Table 1:** Effect of *Agrobacterium* co-cultivation of callus at different ages after its initiation on survival frequency at different stages of *in vitro* culture in two maize genotypes

Age of callus	Genotype	Subculture	Selection-I	Selection-II	Regeneration
**0 days**	NM74C	42.00 ± 7.00	4.66 ± 1.52	1.00 ± 1.00	0.00 ± 0.00
	NM5884	52.66 ± 3.51	15.66 ± 2.08	9.66 ± 2.08	4.33 ± 0.57
**10 days**	NM74C	48.5 ± 5.77	9.28 ± 4.04	1.42 ± 0.57	0.00 ± 0.00
	NM5884	67.00 ± 4.93	33.00 ± 5.29	16.75 ± 2.51	7.50 ± 1.73
**20 days**	NM74C	66.0 ± 6.35	30.17 ± 2.88	4.31 ± 2.88	1.20 ± 0.50
	NM5884	82.63 ± 2.08	67.63 ± 3.04	45.69 ± 8.02	25.13 ± 6.80
**30 days**	NM74C	54.33 ± 6.50	12.66 ± 2.30	2.50 ± 1.00	1.06 ± 0.00
	NM5884	81.00 ± 5.29	57.17 ± 6.02	38.66 ± 4.72	18.83 ± 1.52
**40 days**	NM74C	46.8 ± 5.85	9.83 ± 2.08	2.16 ± 0.57	0.00 ± 0.00
	NM5884	75.33 ± 7.37	43.33 ± 7.63	26.50 ± 5.29	9.66 ± 1.52

Though the IEs are mostly affected with *Agrobacterium* co-cultivation and influenced the survival frequency of explants, calli growth and also regeneration, in order to enhance the survival frequency at every step to achieve maximum frequency of genetic transformation, several parameters involved in the process of transformation have been considered and further studied using the inbred line NM5884, which was found to be more suitable when compared with NM74C.

### Optimization of various factors affecting plant transformation

#### Influence of bacterial population in co-cultivation

Population of bacteria—*i.e.*, concentration of *Agrobacterium tumefaciens* (density) in the infection medium—greatly influences the survival frequency of cultures at each step of the *in vitro* process. Selection and regeneration frequency of E.callus of maize varies with bacterial culture measured at 580 nm. At lower concentration, decreased transformation frequency was observed, and at higher concentration, overgrowth of bacterial culture was observed, and explants turned to necrotic. In the present study, the influence of three different concentrations *viz*., 0.6, 0.8, and 1.0 OD at 580 nm during logarithmic growth stage on transformation efficiency of E. calli were evaluated. Bacterial cells were harvested when the culture attains the optical density of 0.6, 0.8, and 1.0 and used for co-cultivation. Among these concentrations, IEs co-cultivated with the cultures having 0.8 OD resulted in maximum survival frequency of callus at subculture stage (87%), two levels of selection stages (57 and 37% ) as well as regeneration stage (23%) (Table 2). On the other hand, 0.6 OD showed very low survival rate at selection (17%) and regeneration (9%) stages, whereas 31% and 20 % of frequencies were observed in selection and regeneration frequencies respectively with 1.0 OD (Table 2).

**Table 2:** Survival frequency of *in vitro* cultures at three different stages and their frequency of regeneration in maize affected by different parameters with different variables involved in *Agrobacterium*-mediated transformation after co-cultivation

Parameter	Variable	Subculture	Selection-I	Selection-II	Regeneration
**Optical density of bacterial culture**	0.6	60.17 ± 5.13	28.59 ± 4.04	17.19 ± 3.51	8.94 ± 1.00
	0.8	86.93 ± 4.50	57.41 ± 7.76	37.09 ± 4.72	23.38 ± 3.51
	1	73.55 ± 9.50	46.00 ± 3.60	30.66 ± 5.29	19.55 ± 6.50
**Strength of the infection medium**	Half MS (plain)	87.93 ± 2.00	49.48 ± 5.50	40.51 ± 3.51	21.89 ± 3.51
	Full MS (plain)	83.20 ± 5.56	36.98 ± 8.08	28.11 ± 6.80	16.60 ± 3.78
	LS inf	74.92 ± 6.65	23.07 ± 5.00	14.46 ± 6.11	12.30 ± 5.03
**pH of the infection medium**	pH 5.2	50.00 ± 8.88	30.00 ± 2.64	18.88 ± 2.64	13.70 ± 2.08
	pH 5.4	70.30 ± 6.65	40.61 ± 2.64	29.69 ± 5.03	18.61 ± 2.08
	pH 5.8	82.57 ± 5.77	60.60 ± 8.50	39.84 ± 3.78	21.51 ± 5.03
**Acetosyrengone concentration**	0 μM	50.28 ± 4.16	33.71 ± 1.15	17.71 ± 3.05	9.71 ± 1.15
	100 μM	84.21 ± 5.68	63.28 ± 7.00	38.59 ± 2.51	23.59 ± 2.08
	200 μM	73.40 ± 2.51	56.13 ± 6.42	28.40 ± 5.50	18.86 ± 4.50
**Macerozyme treatment**	0.1%	57.16 ± 5.13	36.16 ± 3.78	21.33 ± 5.03	12.50 ± 3.00
	0.2%	42.83 ± 4.04	20.50 ± 6.55	17.00 ± 3.46	10.66 ± 2.51
	0.5%	28.83 ± 7.50	12.33 ± 6.42	10.16 ± 5.13	6.33 ± 5.03
**Vacuum infiltration**	0 min	83.33 ± 7.63	46.16 ± 4.17	34.16 ± 3.51	17.00 ± 2.00
	10 min	61.42 ± 9.07	33.28 ± 3.78	22.71 ± 4.58	11.28 ± 1.52
	15 min	58.62 ± 5.77	28.79 ± 2.08	16.72 ± 2.51	7.93 ± 1.15
**Infection time**	10 min	80.32 ± 7.63	54.91 ± 7.63	38.68 ± 5.50	22.78 ± 5.13
	15 min	70.66 ± 5.29	44.22 ± 3.21	22.44 ± 5.13	17.11 ± 0.57
	30 min	58.97 ± 5.00	33.33 ± 3.00	12.99 ± 5.03	6.32 ± 0.57
**Co-cultivation period**	2 days	84.00 ± 3.60	52.00 ± 9.16	36.66 ± 1.15	27.66 ± 1.52
	3 days	66.33 ± 9.01	36.66 ± 8.50	23.00 ± 3.46	15.83 ± 2.51
	4 days	30.65 ± 5.00	13.69 ± 1.73	15.00 ± 2.00	7.17 ± 1.00

#### Effect of infection medium/ *Agrobacterium* resuspension medium

*Agrobacterium* population, after reaching the density of 0.8 at 580 nm, was centrifuged at 6000 rpm at 4 °C for 20 min to get the sediment cells of bacteria. It was resuspended in infection medium, for which three types of media *viz.*, half MS, full MS and LS media have been used. The concentration of ions present in the medium influences the rate of infection and survival of the bacterial cells. Among the three media used in this study, half MS was found to be the best one for suspending the bacteria for co-cultivation. When the full LS medium was used, the explants became brown and resulted in very low survival frequency on selection medium II (14%) and regeneration medium (12%); on the other hand, MS medium supports the survival rates better than the LS medium; however, the maximum frequency of survival and transformation was observed more with half MS *viz*., 41% and 22% respectively when compared with the full MS medium (Table 2).

### pH of the infection medium

Hydrogen ion concentration (pH) of the infection media used for co-cultivation is an equally important factor in transformation efficiency. pH of infection medium at 5.2, 5.4, and 5.8 were used in co-cultivation and the survival frequencies of callus before transferring to selection medium, on selection media I and II and frequency of regeneration were studied. Among the three pH used, at pH 5.2, survival frequencies of cultures were found to be minimum at all stages (19% on selection medium II, 14% on regeneration). A pH of 5.8 was found to be very suitable for co-cultivation and resulted in maximum survival frequencies on selection (40%) as well as regeneration (22%) stages (Table 2).

### Influence of acetosyrengone concentration

Acetosyringone supplementation in the co-cultivation media influences the survival frequency of culture, which enhances the infection frequency during co-cultivation; in turn, it is also essential for successful transformation. In the present study, two concentrations of acetosyringone *viz.*, 100 and 200 μM were evaluated. A concentration of 100 μM was found to be effective in yielding maximum survival frequencies of cultures and regeneration (39% and 24% respectively) (Table 2). In the contrary, 200 μM concentration resulted in comparatively lower rates of survival frequencies than 100 μM acetosyringone. Infection media without acetosyringone which acts as control were also found to be less effective by showing the survival frequencies of 18 and 10% of callus on selection and regeneration media respectively (Table 2).

### Effect of pretreatment with macerozyme solution

Macerozyme treatment helps the bacteria to penetrate into the cells by digesting the cell wall of the explant so that the penetration of the T-DNA and the vir-genes becomes easy which results in the high frequencies of transformation. Pretreatment with macerozyme was performed with different concentrations *viz.*, 0.1, 0.2, and 0.5% for 5 min before infecting with bacteria. Macerozyme effect was negatively correlated with the survival frequencies of cultures at all stages *i.e.*, survival frequency of callus on selection and regeneration media were found to be decreased with increasing treatment concentration of macerozyme (Table 2). Among the three concentrations used, 0.1% was found to be the best, resulting in 21% and 13% of survival frequencies on selection and regeneration media respectively. Explants treated with macerozyme showed necrosis after a few weeks and failed to develop further in all concentrations; hence, the treatment has not been considered further.

### Influence of vacuum infiltration for the explants

Vacuum infiltration is another way to enhance the frequency of transformation. Hence, in the present study, time duration of vaccum pressure was standardized. A radical decrease in the efficiency of transformation was observed compared with control in which no vacuum infiltration was applied (Table 2). The survival frequency was higher where no vacuum treatment was applied. Survival frequencies (34% and 17%) were found in the selection and regeneration stages respectively. In the contrary, the 10-min vacuum infiltration has given a 23% selection frequency and 11% regeneration frequency. However, the least frequency was observed in the 15-min time duration of 17% and 8% (Table 2).

### Effect of infection time

The effect of infection time on transformation frequency has been studied by using different time periods *viz.,* 10, 15, and 30 min, of which the 10-min time duration has given a maximum selection (39%) and regeneration (23%) frequencies; as time duration increases, the frequency was decreased. Prolonged infection time adversely affected the explant because of bacterial overgrowth. The minimum frequency was observed in 30 min time duration for selection (13%) and regeneration (6%) (Table 2).

### Influence of co-cultivation period

After infecting the cultures with *Agrobacterium*, they were transferred onto the medium supplemented with acetosyrengone at a concentration of 100 μM and incubated in dark for different time periods *viz.*, 2, 3, and 4 days. Survival frequency was found to be maximum in those cultures which were incubated for 2 days and resulted in 37% and also maximum regeneration frequency of 28%. On the other hand, both survival as well as regeneration frequencies were found to be decreased with the increase in incubation period (Table 2).

**Fig. 3:**
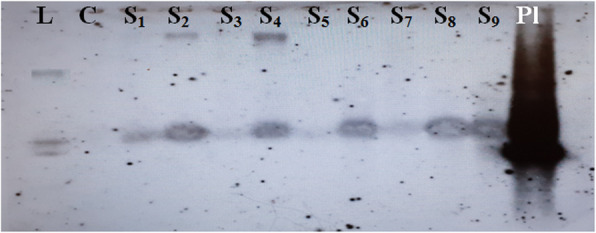
Molecular characterization of putative transgenic maize plants regenerated after *Agrobacterium*-mediated transformation through southern blot analysis carried out using gene (*nced*)-specific probe through non-radioactive labeling method. *C* control, *Pl* plasmid, *L* ladder, *S1–S9* samples 1–9

Immature embryos of NM5884 inbred line were inoculated in larger numbers by adopting the conditions which were found to be suitable and yielded higher frequencies of the survival of cultures and regeneration to evaluate the frequency of transformation which was found to be 6% (Table 3). Putative plants that survived on the selection media were subjected to molecular characterization through testing with PCR by using marker (hygromycin)-specific primers and gene-specific markers *viz*., *rpk* and *nced*. Putative transgenics which were found to be positive in PCR and show good agronomical characters with good seed set have been considered as elite transgenic lines. Three such lines were selected, and seeds were collected from these elite transgenic lines (T_0_) *viz*., ET-2, ET-5 and ET-9, which were forwarded to T_1_ generation. Twenty plants raised from each of these three lines (*i.e*., ET-2-1 to 20, ET-5-1 to 20, and ET-9-1 to 20) were used for screening with PCR. Fifteen plants, five plants from each of the three lines, which were positive in PCR screening, have been used for physiological and biochemical characterization. Out of these 15 plants, nine plants, three plants from each of the three lines after physiological and biochemical analyses have been subjected to southern analysis using the probe designed for *nced* gene by adopting non-radioactive labeling method, to assess the number of inserts in the transgenic maize plants. The resulted transgenic plants were found to have 1–2 copies of genes (Fig. 3).

**Table 3:** Transformation frequency obtained on hygromycin regime and molecular screening of putative transgenics through PCR using *hpt II* primers of maize obtained through *Agrobacterium-*mediated transformation

No. of immature embryos co-cultivated	Survival frequency at subculture stage	Survival frequency in Selection-I	Survival frequency in Selection-II	Regeneration frequency	PCR +ves for ***hpt II***	Transformation frequency (%)
5697	86.29 (4916)	71.19 (4056)	46.58 (2654)	29.1 (1658)	329	5.77

Numbers in parenthesis indicate total number of calli tested

### Physiological and biochemical characterization (*in vitro*)

**Fig. 4:**
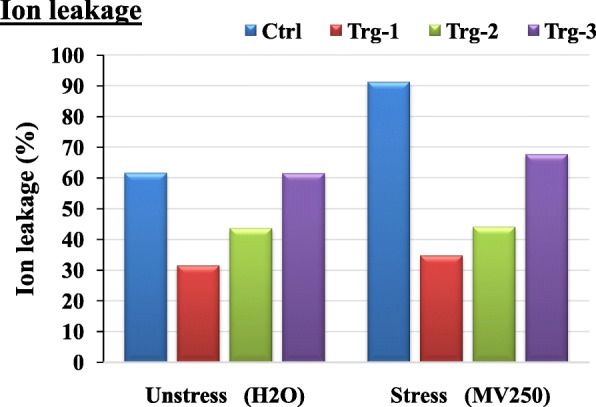
Physiological characterization of putative transgenic maize plants (Trg1–3) regenerated after *Agrobacterium*-mediated transformation and their corresponding untransformed control plants through ion leakage by studying electrical conductivity under normal condition (unstressed) and after giving simulated stress with methyl viologen

**Fig. 5:**
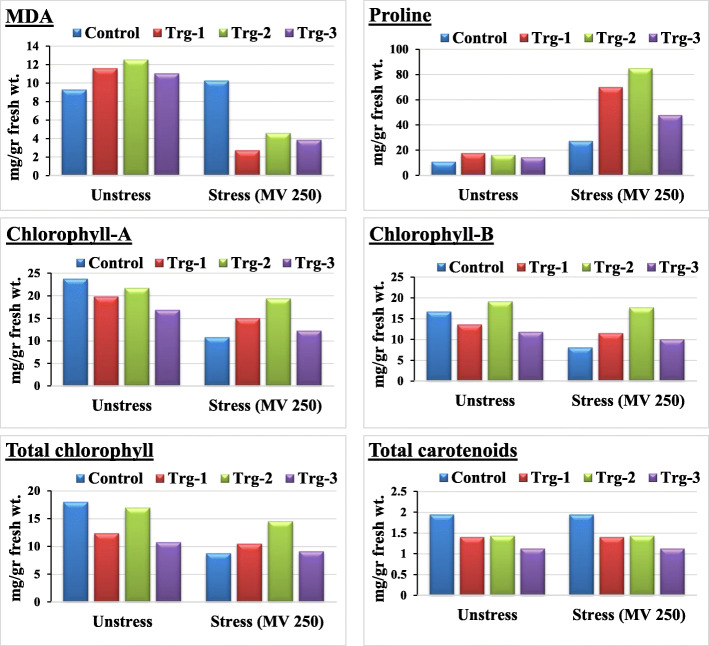
Physiological and biochemical characterization of putative transgenic maize plants (Trg1–3) regenerated after *Agrobacterium*-mediated transformation and their corresponding untransformed control plants through lipid peroxidation, proline estimation, pigment analyses by studying chlorophyll-A, chlorophyll-B, and total chlorophyll and total carotenoids under normal condition (unstressed) and after giving simulated stress with methyl viologen

Prior to the experiments for biochemical and physiological characterization, effective concentration of methyl viologen and treatment time have been established by simulating the stress by using different concentrations *viz*., 50, 100, 150, 200, 250, 300, 350, 400, 450, and 500 μM, for 12-, 24-, and 36-h intervals using the non transgenic plants (control). At the end of the treatment, effective concentration and time were evaluated by considering the degree of damage caused in the pigment quantities under the stress. The 250-μM methyl viologen treatment for 36 h was found to be effective for screening the transgenics. The same treatment concentration and time have been used for all experiments designed for physiological and biochemical characterization, for which ion leakage, lipid peroxidation, proline accumulation and analyses of photosynthetic pigments *viz*., chlorophyll-A, chlorophyll-B, total chlorophyll, and total carotenoids were studies in three elite lines *viz*., ET-2, ET-5, and ET-9, represented as Trg-1, 2, and 3 respectively. In each line, five plants were analyzed. For all these experiments, non transgenic maize plants were served as control plants. All the physiological and biochemical studies were studies in transgenic and control plants under both stressed and unstressed conditions. In all the experiments done *in vitro*, elevated levels of biochemical activity in transgenics over the control plants that were not exposed to methyl viologen were shown, to mitigate the stress for surviving. Since most of the symptoms have been related to membrane damage, experiments have been conducted as a function of electrical conductivity (EC) in the medium where the stressed and unstressed leaf discs of transgenics and control plants have been grown. Significant increase in ion leakage as a function of electrical conductivity has been observed between stressed and unstressed controls; on the other hand, no significant differences have been observed between any of the transgenics (Fig. 4). Membrane damage has also been studied biochemically through lipid peroxidation by measuring the MDA (malondialdehyde) levels in stressed and unstressed controls and transgenics plants (Fig. 5). MDA content in control was found to be increased in stressed condition, whereas in transgenics, MDA-reactive substances were observed to be decreased under stress (Fig. 5). Proline accumulation studies revealed that though both control as well as transgenic plants accumulated more proline under stress than their corresponding counter plants under normal (unstress) conditions, accumulation of proline in transgenic plants was significantly more than in control plants. Among the transgenics, Trg-2 (ET-5) accumulated more proline (Fig. 5). Analysis of photosynthetic pigments *viz*., chlorophyll-A, chlorophyll-B, and total chlorophylls and carotenoids revealed that except carotenoids, chlorophyll-A, chlorophyll-B, and total chlorophyll were found to be decreased or remain unchanged under stress condition in transgenics when compared with normal (unstress) condition, whereas in control plants, decrease in pigment content were more in stress conditions. On the other hand, carotenoid content was found to be unchanged or slightly increased in all transgenics; whereas in nontransgenics, pigment content was found to be unchanged or slightly decreased (Fig. 5).

### Pot experiment of transgenic maize for drought screening (*in vivo*)

**Fig. 6:**
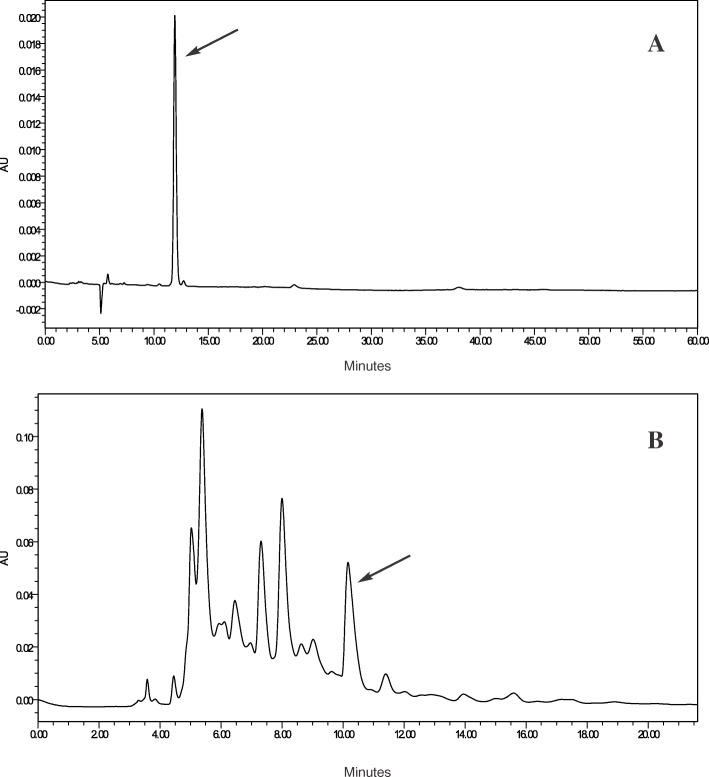
Chromatograms of standard solution of ABA (**A**) and extract of maize plant (**B**) where the arrows represent the ABA peak in both pictures with retention time of ~ 11 min

**Fig. 7:**
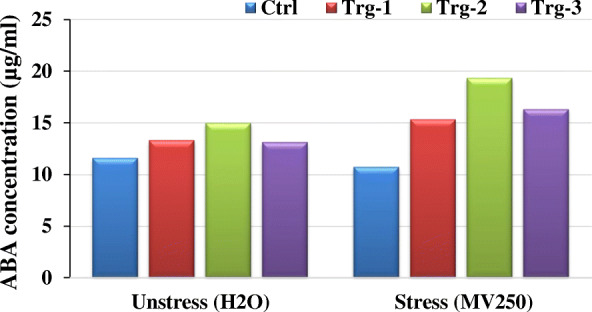
Biochemical characterization of putative transgenic maize plants (Trg1–3) regenerated after *Agrobacterium*-mediated transformation and their corresponding untransformed control plants through ABA enzyme assay under normal condition (unstressed) and after giving simulated stress with methyl viologen

**Fig. 8:**
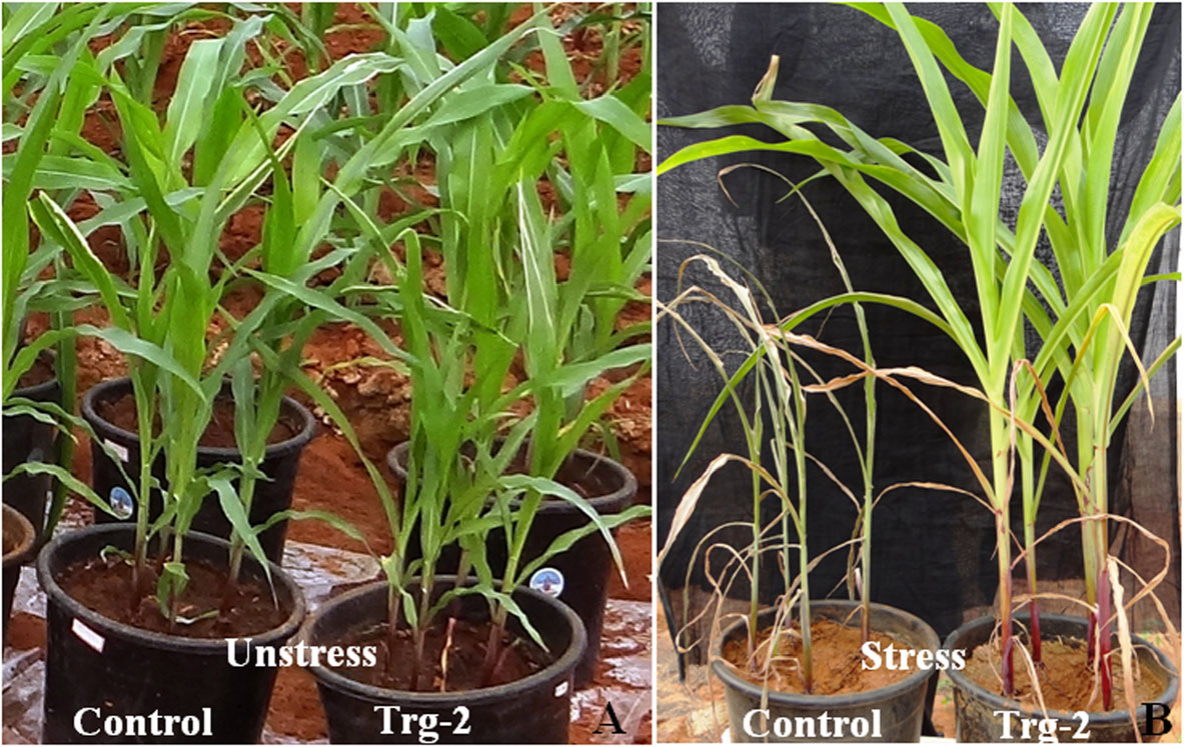
Wild-type and transgenic lines (Trg-2) under well water (**A**) and 14 days of water withholding condition (**B**), tolerant green transgenic (right in **B**), and completely dried non-transgenic (left in **B**) maize plants under drought stress given in pot experiment at the end of 14 days

In order to screen the transgenics in poly house, pot experiments were conducted with three independent transgenic plants (ET-2-3, ET-5-17, and ET-9-14) along with wild-type control under severe water deficit condition (9 days). After 9 days, plants were recovered by pouring the water equally to all plants. Leaf samples were collected and subjected to enzyme assay for ABA. ABA content was measured using the HPLC method, for which standards have been run, retention time (RT) identified (Fig. 6A), and the standard curve prepared. Then the samples, controls, and transgenics were analyzed by comparing with the standard chromatograms (Fig. 6A, B), and results have been presented in Fig. 7. ABA content was found to be increased in all transgenics under stress condition when compared with their corresponding counters in the unstress condition, whereas the control plants showed decreased ABA content under stress condition (Fig. 7). Among the transgenic plants, Trg-2 (ET-5) showed more accumulation of ABA than the other two events *viz*., Trg1 and Trg3 (ET-2 and ET-9). The transgenic plants exposed to drought in pot experiments (*in vivo*) showed significant survival capacity over the control plants. They were left for 14 days, where the control plants under stress (without water) were completely dried and transgenics were left green (Fig. 8). Among the transgenic plants, Trg-2 (ET-5) was found to be more tolerant than the other two based on *in vitro* (physiological and biochemical) and *in vivo* (pot experiment) screenings.

## Discussion

Genes involved in the biochemical pathways which control the reactive oxygen species in the abiotic stress conditions have been introduced and over expressed in the crop plants to develop the stability even in the drastic multiple stress conditions *viz*., drought, water logging, and very high and low temperatures. Many of such abiotic stress-related genes have been isolated and characterized in many of the crop plants [29, 30]. The regulatory components of the drought response have also been identified [31, 32]. Transgenics in different crop plants were developed through up-regulating the general stress response and also the specific physiological pathways which were found to be involved in the stress management processes [33, 34]. Most of the genes involved in drought stress tolerance will be influenced by the ABA pathway, and hence, the genes within the ABA pathway or other genes that alters that pathway will influence the drought tolerance [35]. In the present study, two genes *nced* and *rpk* genes which involved in the ABA pathway have been introduced into maize. Sreenu *et al*. [36] introduced five genes *viz*., *sod*, *apx*, *gr*, *dhar*, and *mdhar*, which play key roles in the ascorbate–glutathione pathway by using *Agrobacterium*-mediated transformation in maize to develop drought-tolerant plants. Li *et al*. [37] developed tomato transgenics with *dhar1* and *dhar2* genes, where *dhar1* showed increased ascorbate content and DHAR activity in both the leaves and fruits. Likewise, in tobacco, transgenic plants were developed with antioxidative enzymes coding genes singly and in combination *viz*., *dhar* or *gst*, or combination of *dhar:gr* and *gst:gr* [38]. Eltelib *et al*. [39] reported higher amount of ascorbate (AsA) in transgenic tobacco plants generated by *mdhar* gene isolated from *Malpighia glabra* L. During pot experiment (dehydration stress), non transgenic control plants showed wilting and complete drying after exposing to stress for long time, whereas transgenic lines survived without any wilting. Performance of transgenic plants (ET-2, ET-5, and ET-9) was relatively commendable in terms of several growth as well as biochemical and physiological parameters in comparison with their respective non-transgenic control lines during simulated drought stress condition. By considering *in vitro* as well as *in vivo* studies together, the over expression of the two genes *viz*., *nced* and *rpk* resulted in good stability in stress and increased the tolerance positively.

As maize is an important crop with a wide range of utilization as food and fodder, extensive exploration of suitable conditions and materials for the development of the crop has been done. For *in vitro* studies and transformation, almost all the plant parts have been used *viz*., immature tassels [40], shoot tips [41, 42], germinated split seeds [43], mature embryos [44], and coleoptile nodes [36, 45]. Among all the explants of maize used, immature embryos were found to be the best explants in most of the studies [46, 47]. In IEs, age and stage were crucial points in yielding high frequencies of E.callus and regeneration and hence different studies reported the optimal conditions and stage of IEs in different genotypes [48, 49]*.*

Infecting the explants with *Agrobacterium* is an initial step in transformation; Sreenu *et al*. [36] reported that infecting the explants (coleoptilar node) with *Agrobacterium* was a suitable method rather than infecting the E.callus in NM81A maize inbred line, where the antagonistic effect was noticed when infection was given to callus. In case of immature embryos, the E. calli especially type II was infected with *Agrobacterium* and reported a high frequency of genetic transformation [50]; on the other hand, Ombori *et al*. [51] gave infection to the IE’s (explants). In the present study, infection has been given to the explants (IEs) which resulted in the browning of explants and finally death without further growth. Hence, the explants were subjected to infection after callus initiation at different time periods. This discrepancy indicated that the genotype plays the role in withstanding for infection to results in high frequencies of transformation. In maize inbred line NM5884, which was used in the present study, Sreenu *et al*. [52] reported the transient expression of GUS, where the E. calli were subjected to microprojectile bombardment, where no infection/co-cultivation step is involved, rather than explant. Such discrepancies have also been noticed in the reports of Saker *et al*. [53] in rice and Tadesse *et al*. [54] in sorghum, where the calli and explants were bombarded respectively. Hence, while doing the transformation, not only genotype and explants but also the method used to introduce the T-DNA has to be considered to achieve the high frequencies of transformation, because in the same genotype (NM5884), when the explants were infected with *Agrobacterium,* no further growth was observed and became necrotic; on the other hand, infection given to IEs after 20 days inoculation resulted in a high frequency of transformation. In the contrary, vice versa results were observed with microprojectile bombardment [52].

Media and its components play important role for *in vitro* growth and regeneration, mostly MS media was used for different explants. Abbas *et al*. [55], Sreenu *et al*. [36], and Pavan Kumar *et al*. [45] established the *in vitro* cultures and *Agrobacterium*-mediated transformation protocols by using MS medium with coleoptilar nodal explants in maize. In the present study, the LS medium has been used and found to be more suitable for the immature embryos, which has also been reported in earlier studies [16, 19].

Genotype is also critical and associated with the explant for *in vitro* growth and regeneration. Sridevi *et al*. [19] reported that inbred line NM5884 is suitable for using IEs as explants, whereas another inbred line NM74C has been reported suitable for using coleoptilar node as an explant [45]. In the present study, it has been further confirmed that NM5884 is the most suitable genotype for using IEs not only for *in vitro* studies but also for genetic transformation in maize.

In the process of *Agrobacterium*-mediated transformation, every step is crucial and specific for genotype and explants used; hence, by varying every parameter, growth and regeneration efficiency was studied at pre-selection and selection stages as well as at the regeneration stage. Pre-culturing of explants before infection is necessary for some genotypes, where the explants did not respond without pre-culturing. Sreenu *et al*. [36] reported 24 h of pre-culturing of explant resulted in best results than the other explants given by no pretreatment and longer (48 h.) pretreatments. Wu *et al*. [56] and Takavar *et al*. [57] reported similar results in wheat and maize respectively. In the present study, culturing of the explants for a longer period (20 days) has a positive response after induction of the callus. Other parameters that involve and influence the transformation frequency were optimized by several workers by using different explants in different genotypes. Present results have similarities as well as differences with their reports which again confirmed that the conditions are dependent on the genotype, explants, and method of transformation used [36, 49].

## Conclusions

In maize, development of transgenic plants through *in vitro* pyramiding of different genes involved in the biochemical pathways that regulates different physiological activities in regulating the multiple stresses in plants is very essential. In this sequence, optimization of conditions and various parameters of transformation improve the transformation frequency. In the present study, introducing two genes involved in the ABA pathway, using gateway technology is useful for developing stable transgenic plants with desired characters (*i.e.*, abiotic stress tolerance). This study also provided the optimized conditions and parameters of *Agrobacterium*-mediated transformation to yield maximum frequency of transformation within NM5884 inbred line.

## Data Availability

Not applicable.

## Abbreviations

2, 4-D: 2, 4-Dichlorophenoxy acetic acid
ABA: *Abscisic acid*
ANOVA: Analysis of variance
BAP: 6-Benzylaminopurine
C-TAB: Cetyltrimethylammonium bromide
DAP: Days after pollination
DNA: Deoxyribonucleic acid
E.callus: Embryogenic callus
*hpt II*: Hygromycin phosphotransferase gene
IEs: Immature embryos
LB: Luria Bertani broth
*nced*: Nine*-*cis*-*epoxycarotenoid dioxygenase
*rpk*: Receptor-like protein kinases
T-DNA: Transfer DNA
